# Mending the medicines supply chain

**DOI:** 10.2471/BLT.20.020320

**Published:** 2020-03-01

## Abstract

Weakness in the global supply chain for injectable medicines is hampering the delivery of care. Lynne Eaton reports.

When Dr. Ian Henderson (name changed at his request) saw that he was down to his last 50 vials of injectable magnesium sulphate, he knew he was in trouble.

A family physician, working in a 150-bed rural hospital in South Africa’s Eastern Cape, Henderson is responsible for delivering a wide range of clinical services to a local population of some 130 000 people. In November 2018 he was working on the obstetrics ward.

“We do roughly 180 deliveries a month,” he says, “and among those, we see around 15 cases of pre-eclampsia and two of eclampsia.”

Pre-eclampsia and eclampsia are major causes of maternal mortality and morbidity worldwide. The first-line treatment for the condition is magnesium sulphate injection.

“Even though injectable magsulf comes in big vials, and even IV bags, we now only seem able to get the one millilitre single dose vial,” Henderson says. “And the vials we had back then were about enough to treat three women.”

Repeated requests to be resupplied from government stores went unanswered, and visits to pharmacies and health facilities in the surrounding area proved fruitless.

“A week into November we were really starting to bite our fingernails,” he says.

That a physician in South Africa should be reporting a shortage of a basic essential medicine, and that the medicine in question should be an injectable, comes as no surprise to Lisa Hedman, a medicines access expert at the World Health Organization.

“There are multiple risk factors associated with medicines shortages, ranging from raw material and manufacturing issues to procurement, transport, storage and use,” Hedman says. “And sterile injectable medicines, especially generic versions of those medicines, are exposed to most of them.”

One of those medicines is heparin, a drug that has been the focus of attention recently because of concerns about potential shortages. An anticoagulant that is widely used to treat heart attacks and prevent blood clots, heparin is also routinely used in orthopaedic and cardiac surgery, as well as to coat materials, such as coronary stents that are in contact with blood. 

The raw material for heparin, its active pharmaceutical ingredient, is derived from the intestinal mucosa of pigs. Supplies of this raw material have been affected by a 2019 outbreak of African swine fever, which has entailed the slaughter of some 300 million pigs in China, the world’s biggest pig producer and a major supplier of the active pharmaceutical ingredient to generic heparin manufacturers.

“Decisions about rationing […] heparin need to be made in the public health context of the affected country”

Lisa Hedman

The impact this outbreak will ultimately have on heparin supplies remains to be seen. “A number of regions have a documented shortage and this is concerning,” says Hedman, “but at present, manufacturers and their regulators are reporting short-term rationing rather than long term stock outs.”

Early indications that there may be a problem include the pharmaceutical company Pfizer raising the price it charges for heparin in January 2020 to offset what the company reports to be a 50% increase in the cost of the active pharmaceutical ingredient. Another is a statement by one of the biggest manufacturers of heparin, the German company Fresenius Kabi, which in July 2019 announced that it was limiting allocations of the medicine to buyers, warning that the situation in China was likely to reduce the supply of the active pharmaceutical ingredient for an unknown period.

Hedman is concerned about such moves stating that, “any decisions about rationing or otherwise conserving the use of heparin need to be made in the public health context of the affected country, including the possibility of using other anti-coagulants where they are available and where it is clinically safe and appropriate.”

Any long-term response to the heparin supply issue is likely to include the development of alternatives to the pig-derived raw material. The logic of this is simple: the more sources there are, the less likely it is that an untoward event, such as the outbreak of a virus, will disrupt the entire supply chain. Heparin was historically prepared from cow lung and sheep intestines and bovine heparin is already manufactured in some countries.

However, supply chain strengthening will also require expansion of the production base. China is the leading producer of the raw material for heparin and the bulk of the product is made by just four companies.

There are a couple of reasons for this concentration. One is the presence of a massive pig production industry, the other is low prices charged by these companies.

According to David Gaugh, senior vice president for sciences and regulatory affairs at the Washington DC based Association for Accessible Medicines – which represents the manufacturers and distributors of generic prescription drugs – generics manufacturers have increasingly sourced their raw materials in China not to maximize profits, but as a matter of survival.

Gaugh argues that the manufacturers of injectable medicines are particularly cost conscious because they are obliged to invest in specialized equipment to manufacture their products, while also maintaining the highest standards of quality and cleanliness.

“The commercial viability of low-profit drugs, such as heparin, are of particular concern to our members,” Gaugh says, adding that some have ceased production. “Once the price drops below a profitable level, the number of firms prepared to make them starts to dwindle, further weakening the supply chain.”

Diogo Piedade, Market Access Manager at Medicines for Europe, an association of European generic medicines manufacturers, shares Gaugh’s concerns. “This is not just a question of heparin,” he says. “The current situation is the result of consolidation that's been taking place in the industry as a whole that's driven by immense downward pressure on prices.”

Gaugh and Piedade are not the first people to make the link between low prices and medicines shortages. A recent report by the drug shortages taskforce of the United States Food and Drug Administration notes that generics with a low-profit margin are susceptible to shortages.

“The commercial viability of low-profit drugs such as heparin are of particular concern to our members,”

David Gaugh

The report notes that slightly under half of the 163 drugs studied that were affected by shortages between 2013 and 2017 were both generics and injectables.

So what can be done? For Suerie Moon, Co-Director of the Global Health Centre at the Graduate Institute of International and Development Studies in Geneva, one answer would be to implement ‘fair pricing’, a concept that she and others have been working on for several years.

Fair pricing is generally discussed in relation to excessive medicines pricing and focused on the needs of consumers. However, as Moon explains, the essence of fair pricing is making sure that the needs of both consumers and producers are satisfied in any given transaction.

Moon defines the space between what is affordable and what is reasonably profitable using a price ceiling and a price floor. “For sellers, a reasonable price floor could include costs of research and development, manufacturing, and distribution,” she says. For buyers, Moon proposes a reasonable price ceiling that could include considerations of present and future affordability, value to the individual and health system, and security of supply.

In other words, Moon’s fair price acknowledges the need to pay prices that don’t drive suppliers out of business. “A number of buyers already take this approach to procurement, including the United Nations Children’s Fund, which is careful to maintain several suppliers for the vaccines it buys and conducts its procurement operations accordingly,” Moon says.

Moon is quick to point out, however, that procurement practices and the prices they can result in, are not the only issue that has a bearing on medicines shortages. “There are multiple factors at work here, and they tend vary from medicine to medicine.”

With regard to magnesium sulphate, for example, medicines manufacturing appears not to be the main issue. Says Hedman: “While there are no reported shortages at the manufacturing level, there is a chronic problem with access to this medicine, mainly due to supply chain issues, including poor forecasting, insufficient planning, procurement and distribution.”

It was those problems that came to bear on Ian Henderson’s rural hospital in November 2018. They were problems he could not do much about. But there was something that he could do, and he did it – jumping in his truck and making the ten-hour round-trip to East London, South Africa, his nearest big city. There, he was able to buy enough ‘magsulf’ to cover the needs of his patients for a few more weeks.

“We had five eclampsia patients in November and December, and about 30 pre-eclampsia patients,” he says. “Without the supplies we bought, some of those women would probably have died.”

**Figure:**
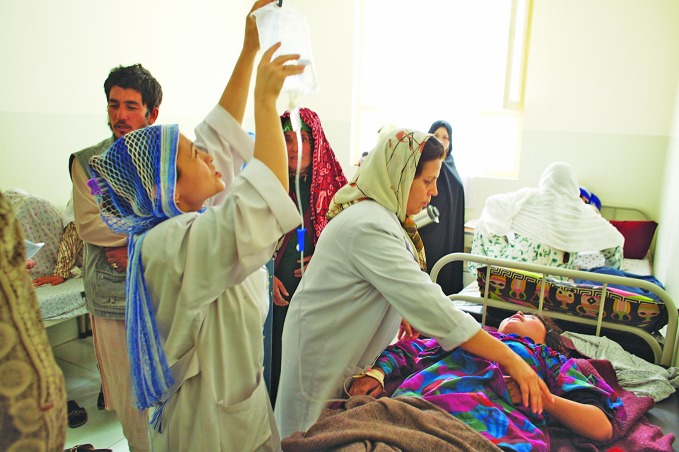
A young woman with pre-eclampsia receives intravenous magnesium sulphate in a clinic in Afghanistan

UNICEF

**Figure:**
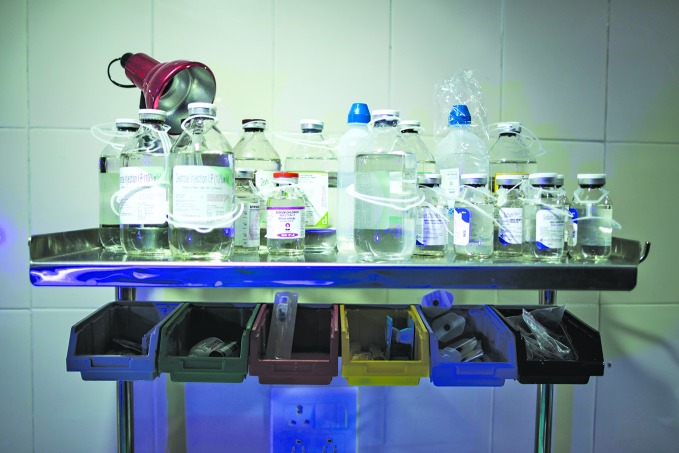
Sterile injectables in a newborn care unit

UNICEF/Vishwanathan

